# Causal association between mTOR-dependent EIF-4E and EIF-4A circulating protein levels and type 2 diabetes: a Mendelian randomization study

**DOI:** 10.1038/s41598-020-71987-8

**Published:** 2020-09-25

**Authors:** Ghada A. Soliman, C. Mary Schooling

**Affiliations:** 1 Department of Environmental, Occupational and Geospatial Health Sciences, The City University of New York, Graduate School of Public Health and Health Policy, 55 West 125th St, New York, NY 10027 USA; 2 School of Public Health, Li Ka Shing, Faculty of Medicine, The University of Hong Kong, 7 Sassoon Road, Hong Kong, China

**Keywords:** Diseases, Risk factors

## Abstract

The mammalian Target of Rapamycin complex 1 (mTORC1) nutrient-sensing pathway is a central regulator of cell growth and metabolism and is dysregulated in diabetes. The eukaryotic translation initiation factor 4E (EIF-4E) protein, a key regulator of gene translation and protein function, is controlled by mTORC1 and EIF-4E Binding Proteins (EIF4EBPs). Both EIF4EBPs and ribosomal protein S6K kinase (RP-S6K) are downstream effectors regulated by mTORC1 but converge to regulate two independent pathways. We investigated whether the risk of type 2 diabetes varied with genetically predicted EIF-4E, EIF-4A, EIF-4G, EIF4EBP, and RP-S6K circulating levels using Mendelian Randomization. We estimated the causal role of EIF-4F complex, EIF4EBP, and S6K in the circulation on type 2 diabetes, based on independent single nucleotide polymorphisms strongly associated (p = 5 × 10^–6^) with EIF-4E (16 SNPs), EIF-4A (11 SNPs), EIF-4G (6 SNPs), EIF4EBP2 (12 SNPs), and RP-S6K (16 SNPs). The exposure data were obtained from the INTERVAL study. We applied these SNPs for each exposure to publically available genetic associations with diabetes from the DIAbetes Genetics Replication And Meta-analysis (DIAGRAM) case (n = 26,676) and control (n = 132,532) study (mean age 57.4 years). We meta-analyzed SNP-specific Wald-estimates using inverse variance weighting with multiplicative random effects and conducted sensitivity analysis. Mendelian Randomization (MR-Base) R package was used in the analysis. The PhenoScanner curated database was used to identify disease associations with SNP gene variants. EIF-4E is associated with a lowered risk of type 2 diabetes with an odds ratio (OR) 0.94, 95% confidence interval (0.88, 0.99, p = 0.03) with similar estimates from the weighted median and MR-Egger. Similarly, EIF-4A was associated with lower risk of type 2 diabetes with odds ratio (OR) 0.90, 95% confidence interval (0.85, 0.97, p = 0.0003). Sensitivity analysis using MR-Egger and weighed median analysis does not indicate that there is a pleiotropic effect. This unbiased Mendelian Randomization estimate is consistent with a protective causal association of EIF-4E and EIF-4A on type 2 diabetes. EIF-4E and EIF-4A may be targeted for intervention by repurposing existing therapeutics to reduce the risk of type 2 diabetes.

## Introduction

Diabetes is one of the most prevalent chronic diseases globally and in the US and is associated with several co-morbidities. In 2015, it was estimated that 9.4% of the US population, or approximately 30.3 million people have diabetes^1^. Among those, only 23.1 million people are diagnosed, and about 7.2 million are undiagnosed. Type 2 diabetes represents approximately 90 to 95% of all diabetes cases. The estimated direct medical costs of diagnosed diabetes in 2017 was $237 billion in the US with an average medical expenditure of diagnosed person of $13,700 per year^2^. It is further estimated that 33.9% of US adults have prediabetes. To date, the causes of type 2 diabetes are not fully understood. Diabetes is also associated with multiple co-morbidities and complications, including obesity. Thus, there is an urgent need to determine causal associations and develop new strategies for prevention, early detection, diagnosis, and treatment of type 2 diabetes.

The mechanistic Target of Rapamycin (mTOR) is a highly conserved serine/threonine kinase and a key regulator of cell growth and metabolism^3–5^. As such, mTOR protein is a central metabolic integrator and is dysregulated in type 2 diabetes and diabetes-associated co-morbidities^6–8^. Importantly, mTOR is a druggable protein and therefore is a potential target for type 2 diabetes interventions. It nucleates two functionally-distinct and mutually-exclusive complexes, namely mTOR Complex 1 (mTORC1) and mTOR Complex 2 (mTORC2). Both mTORC1 and mTORC2 regulate cellular metabolism, survival, proliferation, and growth. mTORC1 is a central hub for nutrient-sensing and energy metabolism and, as such, coordinates anabolic protein and nucleotide synthesis and catabolic autophagy^9–16^. On the other hand, mTORC2 drives insulin signaling by phosphorylating Akt (^Ser473^)/PKB downstream of the phosphoinositide 3-kinase (PI3K)/insulin pathway^17–20^.

**Figure 1:**
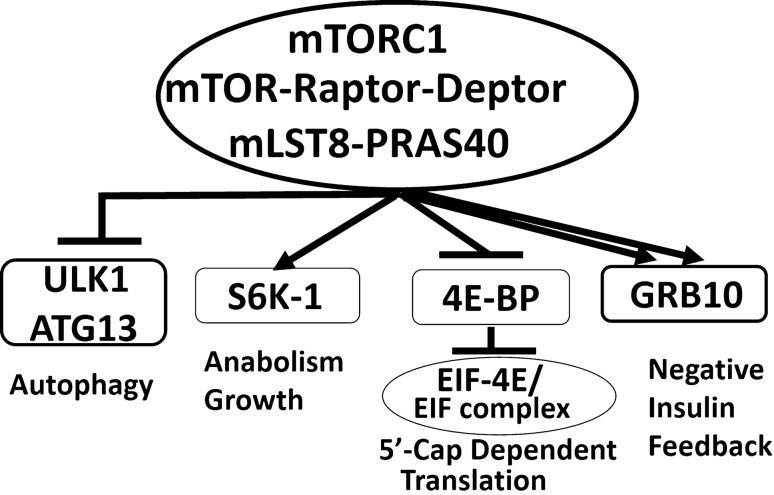
Diagrammatic representation of the mTORC1 complex. mTOR protein kinase nucleates two distinct and mutually exclusive complexes, namely mTORC1 and mTORC2. mTORC1 receives inputs from nutrients, insulin, growth factors, cytokines, and environmental cues; and transmits signals to downstream targets including S6K1, 4E-BP, and EIF complex, GRB10, and ULK1/ATG13.

The mTORC1, which binds exclusively to Raptor protein and other partners (Fig. 1), regulates two downstream effectors, namely, Ribosomal Protein-S6 kinase 1 (RP-S6K 1) and eukaryotic translation initiation factor 4E-binding protein (EIF4EBP). The first mTORC1 target, RP-S6K1 phosphorylates eukaryotic translation initiation factor 4B (EIF-4B), which is a positive regulator of EIF-4F complex^21^. In addition, RP-S6K also phosphorylates the programmed cell death protein 4 (PDCD4), which is a negative regulator of EIF-4A and targets it to degradation via the ubiquitin pathway^22^. The second mTORC1 downstream target, EIF4EBP, is a repressor of the translation initiation complex (EIF-4F), which is required for 5′cap-dependent translation of mRNA (Fig. 1). Once phosphorylated by mTORC1, EIF4EBP dissociates from EIF-4E, allowing for the assembly of the EIF-4F complex and initiation of 5′cap-dependent translation. The EIF-4F complex consists of EIF-4E, EIF-4G, and EIF-4A. EIF-4E binds to 7-methylguanosine cap at the 5′-UTR of eukaryotic mRNA and mediates the recruitment of mRNA on ribosomes to start protein translation. As mentioned earlier, the mTOR kinase attracts different protein partners to generate two functionally distinct complexes, namely, mTORC1 and mTORC2. While mTORC1 integrates inputs from nutrients and growth factors and coordinates cellular growth and metabolism^23–25^; mTORC2 is activated by growth factor signals only and responds by phosphorylating the C-terminal hydrophobic motif of Akt/PKB on serine 473^17,26–29^. As such, mTOR complexes and their downstream targets are actionable proteins and metabolic targets in managing type 2 diabetes and mediate mTOR central role in glucose and energy metabolism and in pancreatic progenitor cell growth. Dysregulation of mTORC1 and mTORC2 complexes is linked to insulin resistance and type 2 diabetes via several reciprocal mechanisms in animal models and cell culture^30–41^. However, the causal association of mTOR pathways with diabetes is difficult to establish using observational studies in humans, and randomized controlled trials (RCTs) would be expensive. The Mendelian Randomization (MR), instrumental variable analysis using genetic variants, provides unbiased and unconfounded estimates by taking advantage of the randomization during meiosis and conception^42^. Studies in animal models and cell culture document a biphasic response of mTOR on glucose and energy metabolism^16,40,41,43,44^. Recently, studies in the conditional knockout mice found that loss of mTORC1 impairs pancreatic beta-cell mass and functions as well as the cap-dependent protein translation^45,46^. Therefore, we hypothesized that the elevated levels of circulating proteins of the downstream signals of mTORC1 could be protective from type 2 diabetes mellitus.

In this study, we investigated whether the risk of type 2 diabetes varied with EIF4EBP2, EIF-4E, EIF-4G, EIF-4A, and RP-S6K levels using the Mendelian Randomization approach. We hypothesized that mTOR downstream effectors are metabolic targets in managing type 2 diabetes due to the mTOR central role in glucose and energy metabolism and in the pancreatic progenitors' cell growth.

## Subjects and methods

### Study design and participant flowchart

**Figure 2:**
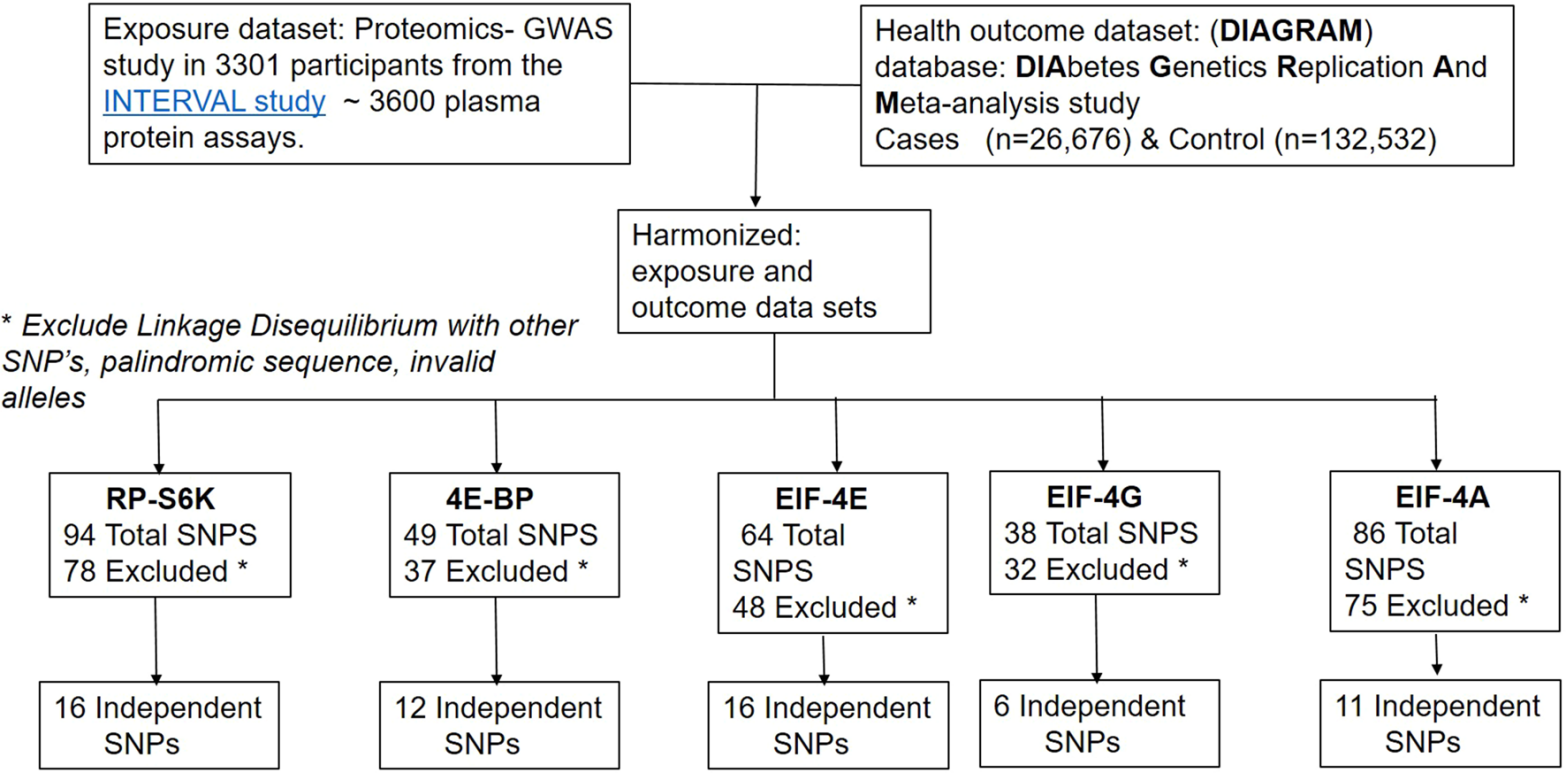
Flow diagram of the selection of SNPs genetic variants as instrumental variables related to exposure harmonized with the dataset for diabetes outcome. We employed a two-sample MR, using summary statistics from two GWAS genetic datasets. For exposure, we used Proteomics-GWAS study^39^. For the health effect outcome, we leveraged the publically available DIAbetes Genetics Replication And Meta-analysis (DIAGRAM) case (n = 26,676), and control (n = 132,532) study (mean age 57.4 years). We applied the genetic predictors of mTOR downstream targets, namely RP-S6K, EIF4E-BP2, EIF-4E, EIF-4A, and EIF-4G, to DIAGRAM genotyped case–control study to determine the causal association between mTOR-targets and diabetes mellitus. Mendelian Randomization (MR-Base) R package was used in the analysis.

We employed a two-sample MR design, where we used aggregate summary statistics from two Genome-wide Association Studies (GWAS) (Fig. 2). For the exposures, we used proteomics- GWAS INTERVAL study^47^. For diabetes mellitus health outcomes, we leveraged the publically available DIAbetes Genetics Replication and Meta-analysis (DIAGRAM) GWAS (Supplemental Table 1). We applied the genetic predictors of mTOR downstream targets, namely RP-S6K, EIF4EBP2, and EIF complexes in the circulation, EIF-4E, EIF-4A, and EIF-4G, to the DIAGRAM genotyped case–control study to determine the causal association between mTOR-downstream targets and type 2 diabetes. The flowchart and the selection process of the data included in the study design are summarized in Fig. 2.

### Approach

#### Genetic prediction of the exposure

Data about mTOR-related gene exposure were obtained from the publically available INTERVAL study Proteomics- GWAS data in 3,301 participants from the INTERVAL study, which included ~ 3,600 plasma protein assay https://www.phpc.cam.ac.uk/ceu/proteins/^47^. The protein concentrations were measured using the SomaLogic method. This SomaScan proteomic assay utilizes a protein-capture Slow Off-rate Modified Aptamer (SOMAmer). The SOMAmer reagents have dual characteristics as protein-affinity binding agents and unique nucleotide sequences recognized by DNA probes for quantification, which enhances the sensitivity of the proteomic assay^48^. Protein concentrations were quantified as relative fluorescent units^47^. The initial INTERVAL study recruited 45,264 healthy blood donors who were 18 years or older and were eligible to donate blood within 25 National Health Service (NHS) Blood and Transplant Centers in England^49,50^. The trial was registered under # ISRCTN24760606, and recruitments were conducted from June 2012–June 2014. We used the publically available datasets.

As mentioned above, in this study, we obtained single nucleotide polymorphisms (SNPs) which strongly (p-value 5 × 10^–6^), and independently (r^2^ < 0.05) predicted RP-S6K, EIF4EBP2, EIF-4E, EIF-4A, and EIF-4G from a GWAS of 3,301 participants in the INTERVAL study^47^. Genetic associations were obtained using linear regression of the natural log-transformed protein abundances adjusted for age, gender, the time between blood collections, processed for biochemical analysis, and scaled to account for population stratification^47,51^. To ensure the SNPs for each exposure were independent (i.e., not in linkage disequilibrium), we used MR Base function "clump data" to select and remove SNPs correlated SNPs.

#### Genetic prediction of health outcomes

Data on diabetes health outcomes were obtained from publically available genetic associations with diabetes from the DIAbestes Genetics Replication And Meta-analysis (DIAGRAM) case–control study https://www.diagram-consortium.org/download. We applied the SNPs predicting the exposures mentioned above to publically available genetic associations with diabetes from the DIAbetes Genetics Replication And Meta-analysis (DIAGRAM) case (n = 26,676), and control (n = 132,532) study (mean age 57.4 years).

### PhenoScanner curated database

The phenoScanner database contains curated large scale GWAS data and facilitates phenome scans, and genotype–phenotype associations across databases to link SNPs with disease biology and mechanistic pathways^51,52^. We used the PhenoScanner to link the identified SNPs in the current study with possible mechanistic pathways of type 2 diabetes mellitus. We inputted the specific SNPs associated with circulating protein levels of interest to determine the associations with disease traits.

### Statistical analysis

We assessed the strength of the genetic instruments from the F-statistic using an approximation, an F-statistics > 10 makes weak instrument bias unlikely (Supplemental Table 2)^53^. To obtain the MR estimates, we used a meta-analysis of SNP-specific Wald estimates from inverse variance weighting (IVW) with multiplicative random effects^54–57^. The SNPs were aligned based on the allele letter and allele frequency. The Wald estimate was calculated as the ratio of the estimate for SNPs on diabetes divided by the estimate for SNPs on exposure for each exposure, i.e., RP-S6K, EIF4EBP2, EIF-4E, EIF-4A, and EIF-4G. SNPs were excluded if they were not in the 1,000 Genomes catalog. We corrected for multiple comparisons by using Bonferroni correction. Mendelian Randomization (MR-Base) R package version 3.6.1 was used in the analysis^58^.

### Sensitivity testing

For the IVW estimate to be valid, all the SNPs should be valid instruments. To establish the validity of the SNPs as genetic instrumental variables (IV), we used the following tests: a weighted median and MR-Egger estimates^59^. The weighted median provides correct estimates when SNPs accounting for > 50% of the weight are valid^60^. MR-Egger adapts the IVW analysis by allowing a non-zero intercept. This balance would allow for the detection of an unbiased causal effect, even if the IV2 assumption was violated. To test for heterogeneity, we used the Cochrane Q statistic, as well as I^2^, which is calculated as 100% × (Q − df)/Q, where Q is Cochran's Heterogeneity statistics, and df is the degrees of freedom^61^.

MR-Egger can give an accurate estimate when the assumption of independent effects are satisfied and that there is little evidence of the pleiotropic effects of SNPs on the outcome (Supplemental Figures 1–3). In addition, if MR-Egger does not correct for unknown pleiotropy, we also used MR-PRESSO, which detects and corrects for outliers^62,63^.

**Figure 3:**
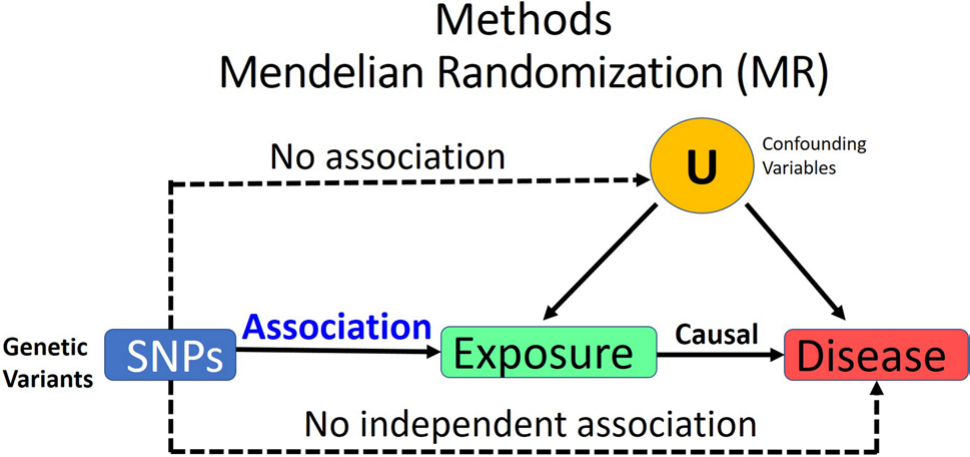
Mendelian Randomization unbiased causal associations and assumptions. Mendelian Randomization (MR) is an application of the instrumental variable using genetic variants Genome-Wide Association Studies (GWAS), and Single Nucleotide Polymorphisms (SNPs). MR requires several stringent assumptions to be fulfilled. First, no confounders are associated with the genetic instrument; and second, the genetic proxy of exposure (SNP) should not be independently associated with the disease outcome, but only mediates its effect via the relevant exposure. The SNPs used in the study had no association with the confounding variables (U) and no independent association with diabetes.

We used the R package "clump_data" to obtain independent SNPs, and the R Package "Mendelian Randomization" and MR-PRESSO to obtain MR estimates. MR-Base analytical platform web application and MR-Base R package were used^64–66^. MR-Base web applications were used to generate some of the graphs, URL: https://www.mrbase.org/. We leveraged R (version 3.6.2). R language and environment for statistical analysis were used for all analyses (URL: https://www.R-project.og/) Foundation for Statistical Computing, Vienna, Austria. Mendelian Randomization requires several stringent assumptions to be fulfilled (Fig. 3). First, no confounders are associated with the genetic instrument; second, the genetic proxy of exposure (SNP) should not be independently associated with the disease outcome, but only mediates its effect via the relevant exposure. The SNPs used in the study had no association with the confounding variables (U), and no independent association with diabetes.

### Ethics

We conducted secondary analysis from publically available aggregate summary data with no involvement of the participants in the primary studies. No original data were generated from this manuscript. Ethical approval of each of the studies used is available in the original publications. There is no required IRB for the secondary analysis of summary data. This study follows the ethical guidelines of the Declaration of Helsinki 1975.

## Results

As mentioned earlier, for genetic prediction of exposure, we obtained SNPs that strongly and independently predicted RP-S6K, EIF4EBP2, EIF-4E, EIF-4A, and EIF-4G circulating plasma levels from a GWAS of 3,301 participants in the INTERVAL study^47^. For the genetic prediction of diabetes outcomes, we applied these SNPs predicting the exposures to publically available genetic associations with diabetes from the DIAGRAM case (n = 26,676) and control (n = 132,532) study (mean age 57.4 years). We used the R packages "clump_data" to obtain independent SNPs.

Of the 94 SNPs predicting RP-S6K, 78 correlated SNPs were excluded, with the remaining 16 SNPs that are uncorrelated were included in the analysis. Of the 49 SNPs predicting EIF4EBP2, 37 correlated SNPs were excluded, giving 12 independent SNPs. Of the 64 SNPs predicting EIF-4E, 48 correlated SNPs were excluded, leaving 16 independent SNPs; of the 38 SNPs predicting EIF-4G, 32 correlated SNPs were excluded, resulting in 6 independent SNPs. Finally, of the 86 SNPs predicting EIF-4A, 75 correlated SNPs were excluded, yielding 11 independent SNPs (Fig. 2).

Table 1 shows that EIF-4E cap-dependent translation factor circulating level was associated with a lower risk of type 2 diabetes with an odds ratio (OR) 0.94, 95% confidence interval (0.88, 0.99, p = 0.03) with similar estimates from the weighted median and MR-Egger. Protein concentrations were measured using the SOMAscan assay^47^. Protein concentrations were quantified as relative fluorescent units. Similarly, EIF-4A cap-dependent translation factor was associated with a lower risk of type 2 diabetes with an odds ratio (OR) 0.90, 95% confidence interval (0.85, 0.97, p = 0.0003). The estimates were very similar in sensitivity analysis, although the MR-Egger estimate had wide confidence intervals.

**Table 1:** Mendelian randomization estimates for the association of EIF-4E (based on 16 independent SNPs with the p-value of 5 × 10^–6^), EIF-4A (based on 11 independent SNPs with the p-value of 5 × 10^–6^), EIF-4G (based on 6 independent SNP's with the p-value of 5 × 10^–6^), EIF4E BP2(based on 12 independent SNPs with the p-value of 5 × 10^–6^), and RP-S6K (based on 16 independent SNPs, with the p-value of 5 × 10^–6^) with diabetes, using public summary data from DIAGRAM meta-analysis, http://www.cardiogramplusc4d.org/media/cardiogramplusc4d-consortium/data-downloads/UKBB.GWAS1KG.EXOME.CAD.SOFT.META.PublicRelease.300517.txt.gz, and human plasma proteome (Sun BB et al. Nature 2018^47^). Protein concentrations were quantified as relative fluorescent units.

Exposure	Mendelian randomization method	Odds ratio	95% confidence interval	p-value	Cochran's Q statistic (p-value)	MR-Egger	
						Intercept p-value	I^2^
EIF-4E Effect size	Inverse variance weighted	0.94	0.88, 0.99	0.027	8.397 (0.906)	–	–
	Weighted median	0.91	0.84, 0.98	0.018	–	–	–
	MR-Egger	0.93	0.83, 1.05	0.265	8.392 (0.867)	0.944	83.4%
	MR PRESSO (uncorrected)	0.94	0.90, 0.98	0.009	–	–	–
EIF-4A Effect size	Inverse variance weighted	0.90	0.85, 0.97	0.003	2.662 (0.988)	–	–
	Weighted median	0.90	0.82, 0.98	0.017	–	–	–
	MR-Egger	0.91	0.80, 1.05	0.211	2.628 (0.977)	0.853	88.3%
	MR PRESSO (uncorrected)	0.90	0.87, 0.94	0.0002	–	–	–
EIF-4G3 Effect size	Inverse variance weighted	1.08	0.97, 1.19	0.169		–	–
	Weighted median	1.08	0.95, 1.24	0.252	–	–	–
	MR-Egger	0.92	0.70, 1.20	0.540	1.819 (0.7)	0.213	51.3%
	MR PRESSO (uncorrected)	1.08	0.99, 1.17	0.154	–	–	–
EIF4EBP2 Effect size	Inverse variance weighted	0.96	0.88, 1.05	0.372	16.75 (0.115)	–	–
	Weighted median	1.00	0.90, 1.12	0.973	–	–	–
	MR-Egger	1.20	0.98, 1.48	0.080	10.917 (0.364)	0.021	0.6%
	MR PRESSO (uncorrected)	0.96	0.88, 1.05	0.391	–	–	–
RP-S6K	Inverse variance weighted	0.95	0.89, 1.01	0.077	18.747 (0.225)	–	–
	Weighted median	0.93	0.85, 1.00	0.061	–	–	–
	MR-Egger	0.92	0.82, 1.04	0.194	18.492 (0.185)	0.660	89.5%
	MR PRESSO (uncorrected)	0.95	0.89, 1.01	0.097	–	–	–

The data shows that EIF-4E cap-dependent translation factor was associated with a lower risk of type 2 diabetes with an odds ratio (OR) 0.94, 95% confidence interval (0.88, 0.99, p = 0.03) with similar estimates from the weighted median and MR-Egger. Similarly, EIF-4A cap-dependent translation factor was associated with a lower risk of type 2 diabetes with an odds ratio (OR) 0.90, 95% confidence interval (0.85, 0.97, p = 0.0003). The estimates were very similar in sensitivity analysis, although the MR-Egger estimate had wide confidence intervals. Mendelian Randomization (MR-Base) R package version 3.6.1 was used in the analysis.

EIF-4G3 and EIF4EBP2 were not associated with diabetes with odds ratio (OR) 1.08, 95% confidence interval (0.97, 1.19, p = 0.169), and odds ratio (OR) 0.99, 95% confidence interval (0.86, 1.03, p = 0.09), respectively. Similarly, ribosomal protein S6K was not associated with diabetes, OR 0.95, 95% confidence interval (0.89, 1.01, p = 0.08). Sensitivity analysis gave consistent estimates.

**Figure 4:**
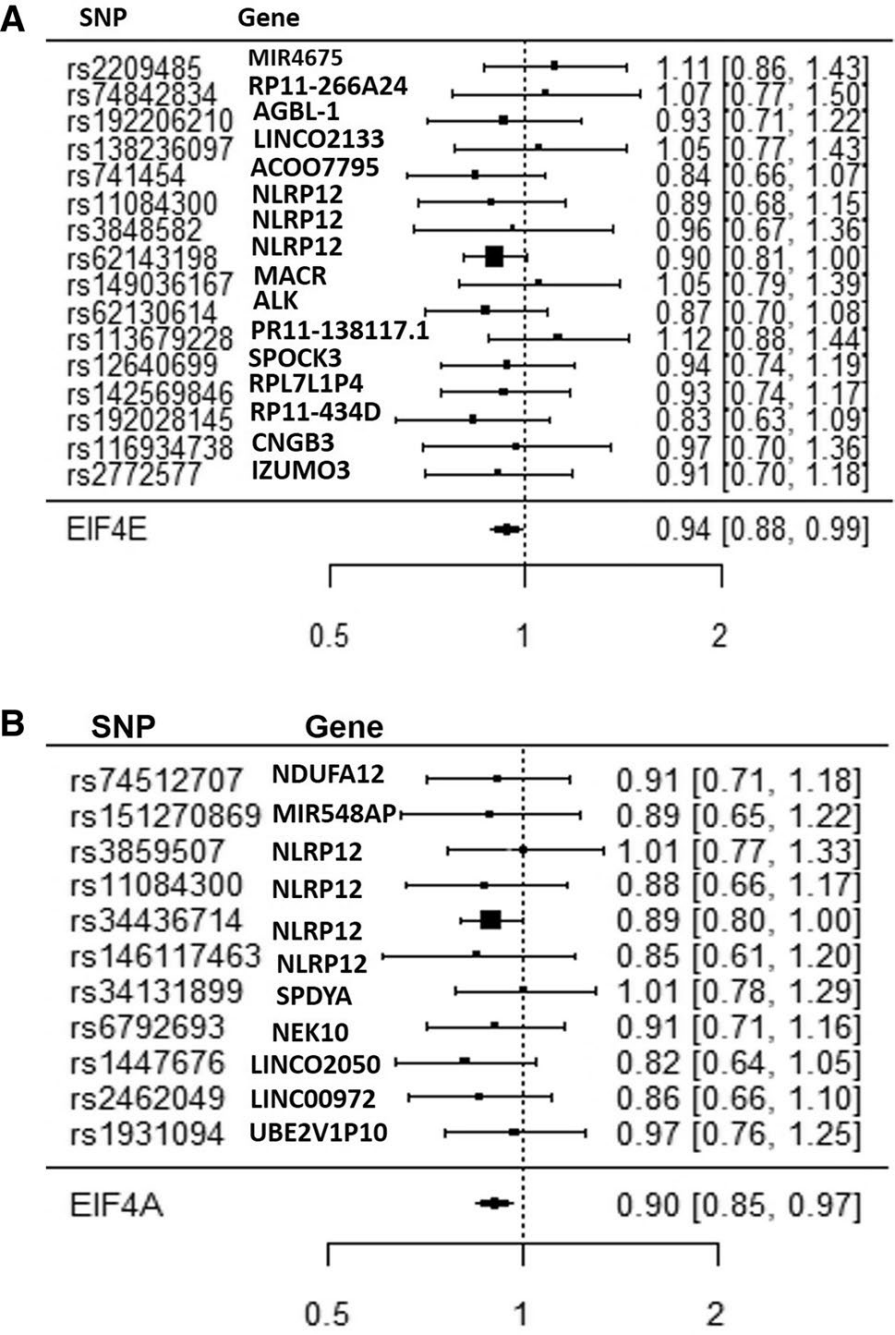
(**A**) SNP variants associated with EIF-4E and the risk of type 2 diabetes. Odds ratio ± 95% confidence interval of the EIf-4E causal estimates on types 2 diabetes are shown. Inverse Variance Weighted (IVW) analysis of individual SNPs and the composite odds ratio of all SNPs shows the independent SNPs that are strongly associated with EIF-4E. Using phenoScanner v2 database of human genotype–phenotype association based on the UKBB data https://www.phenoscanner.medschl.cam.ac.uk, the following 16 Independent SNPs associated with EIF-4E merged with diabetes health outcome. Out of the 16 SNPs, 10 were located in the intron region of the following genes, RP11-266A241, AGBL1, LINCO2133, NLRP12 (3 SNPs), MARCO, ALK, PR11-138117.1, SPOCK3, RP11-434D9.1, CNGB3; 3 SNPs were located intergenic region to the nearest genes MIR4675, ACOO77851, and RLP7L1P4. One SNP was located upstream of the IZUMO3 gene. Mendelian Randomization (MR-Base) R package was used in the analysis^58^. (**B**) SNP variants associated with EIF-4A and the risk of type 2 diabetes. Odds Ratio ± 95% Confidence Interval of Individual SNP causality on type 2 diabetes are shown. Inverse Variance Weighted (IVW) analysis of individual SNPs and the composite odds ratio of all SNPs shows the independent SNPs that are strongly associated with EIF-4A. Using phenoScanner v2 database of human genotype–phenotype association based on publically available databases https://www.phenoscanner.medschl.cam.ac.uk, the following 11 Independent SNPs associated with EIF-4A were harmonized with diabetes health outcome. Out of the 11 SNPs, 5 were located in the intron region of the following genes NDUFA12, SPDYA, NEK10, LINCOO972, and NLPR12; 3 SNPs were located intergenic region to the nearest genes, MIR548AP, LINCO2050, and UBE2VIP10. 1SNP was located upstream of gene NLPR12, 1 downstream NLPR12 gene, and 1 missense on gene NLPR12. Mendelian Randomization (MR-Base) R package was used in the analysis^58^.

The following 16 Independent SNPs were associated with EIF-4E circulating protein level (Table 2) and the harmonized outcomes were characterized, as shown in Fig. 4A. Similarly, we identified 11 independent SNPs associated with EIF-4A circulating levels and associated with protective effects from the risk of diabetes (Table 2). We leveraged the use of the PhenoScanner v2 curated database of human genotype–phenotype associations based on cataloging GWAS publically available data from over 150 million unique SNPs and 65 billion associations https://www.phenoscanner.medschl.cam.ac.uk^51,52^. Out of the 16 SNPs variants genetically associated with EIF-4E, twelve SNPs were located in the intron region of the following genes, RP11-266A241, AGBL1, LINCO2133, NLRP12 (3 SNPs), MARCO, ALK, PR11-138117.1, SPOCK3, RP11-434D9.1, CNGB3; also, three SNPs were located in the intergenic region of MIR4675, ACOO77851, and RLP7L1P4; and one SNP was located upstream of IZUMO3 gene. Most of the SNPs had no known disease associations. However, only three SNPs were associated with NLRP12, a marker of monocytes, and macrophages mediated inflammasome.

**Table 2:** Association of genetically predicted mTOR-downstream targets exposures with diabetes outcome SNPS.

	Chr:position outcome	Beta. outcome	SE outcome	Effect allele	Other allele	p-value outcome	SNP	Beta exposure	SE exposure	Effect allele	Other allele	p-value exposure	Wald	Waldvar
**EIF4E**														
1	10:20905192	0.017	0.021	T	C	0.41	rs2209485	0.1637	0.034	T	c	1.51E−06	0.103849	0.016457
2	11:23,204,504	0.032	0.075	T	G	0.67	rs74842834	0.4443	0.0947	T	g	2.69E−06	0.072023	0.028495
3	15:87118467	0.044	0.081	T	G	0.59	rs192206210	− 0.5846	0.1211	T	g	1.41E−06	− 0.07527	0.019198
4	16:47761066	− 0.014	0.042	A	G	0.73	rs138236097	− 0.2673	0.0583	A	g	4.47E−06	0.052376	0.024689
5	19:29350614	0.025	0.018	T	C	0.16	rs741454	− 0.1426	0.031	T	c	4.17E−06	− 0.17532	0.015933
6	19:54311090	− 0.019	0.021	T	C	0.36	rs11084300	0.1585	0.0272	T	c	5.75E−09	− 0.11987	0.017554
7	19:54317794	0.0077	0.032	A	G	0.81	rs3848582	− 0.1776	0.0364	A	g	1.07E−06	− 0.04336	0.032465
8	19:54320939	− 0.05	0.026	A	G	0.055	rs62143198	0.4669	0.0296	A	g	6.76E−56	− 0.10709	0.003101
9	2:119720418	0.024	0.075	T	C	0.75	rs149036167	0.5168	0.108	T	c	1.70E−06	0.04644	0.021061
10	2:29505824	− 0.065	0.051	T	G	0.2	rs62130614	0.4606	0.0999	T	g	3.98E−06	− 0.14112	0.01226
11	4:138007589	0.04	0.044	A	G	0.36	rs113679228	0.3464	0.0732	A	g	2.24E−06	0.115473	0.016134
12	4:167757172	− 0.01	0.019	A	C	0.6	rs12640699	0.1574	0.0341	A	c	4.07E−06	− 0.06353	0.014571
13	5:116455931	− 0.017	0.027	T	G	0.52	rs142569846	0.2303	0.0468	T	g	8.71E−07	− 0.07382	0.013745
14	5:66,685,975	− 0.098	0.074	A	G	0.19	rs192028145	0.5295	0.1025	A	g	2.40E−07	− 0.18508	0.019531
15	8:87691869	0.011	0.064	T	C	0.87	rs116934738	− 0.3758	0.077	T	c	1.05E−06	− 0.02927	0.029003
16	9:24547232	− 0.013	0.018	A	G	0.46	rs2772577	0.1361	0.0292	A	g	3.02E−06	− 0.09552	0.017492
**EIF-4G3**														
1	12:116387486	0.026	0.015	A	G	0.089	rs7955609	0.1251	0.0263	A	g	2.00E−06	0.207834	0.014377
2	12:127417047	− 0.089	0.1	T	C	0.38	rs112309230	0.5745	0.1158	T	c	6.92E−07	− 0.15492	0.030298
3	12:39641451	− 3.00E−04	0.027	T	C	0.99	rs143862167	− 0.204	0.044	T	c	3.55E−06	0.001471	0.017517
4	17:26694861	0.013	0.015	A	G	0.37	rs704	0.1618	0.0245	A	g	4.27E−11	0.080346	0.008595
5	3:53892603	0.054	0.08	T	C	0.5	rs142978915	0.4055	0.0885	T	c	4.57E−06	0.133169	0.038922
6	9:3762150	0.029	0.057	A	G	0.61	rs1411879	0.4092	0.089	A	g	4.27E−06	0.07087	0.019403
**EIF-4A**														
1	12:95350637	− 0.016	0.023	A	G	0.49	rs74512707	0.1785	0.0385	A	g	3.55E−06	− 0.08964	0.016603
2	15:86409988	− 0.053	0.074	A	G	0.48	rs151270869	0.4543	0.0938	A	g	1.29E−06	− 0.11666	0.026532
3	19:54295370	0.0014	0.025	T	G	0.96	rs3859507	0.1777	0.0327	T	g	5.75E−08	0.007878	0.019793
4	19:54311090	− 0.019	0.021	T	C	0.36	rs11084300	0.1431	0.0272	T	c	1.48E−07	− 0.13277	0.021536
5	19:54327313	− 0.052	0.026	A	C	0.042	rs34436714	0.4687	0.0291	A	c	2.00E−58	− 0.11095	0.003077
6	19:54329672	0.033	0.036	A	G	0.35	rs146117463	− 0.2062	0.042	A	g	9.33E−07	− 0.16004	0.030481
7	2:29058128	− 0.0015	0.036	A	G	0.97	rs34131899	− 0.2829	0.0591	A	g	1.70E−06	0.005302	0.016193
8	3:27245225	0.013	0.017	A	G	0.45	rs6792693	− 0.134	0.0286	A	g	2.82E−06	− 0.09701	0.016095
9	3:74143047	0.024	0.015	T	C	0.11	rs1447676	− 0.1182	0.0257	T	c	4.37E−06	− 0.20305	0.016105
10	7:85099898	− 0.018	0.015	T	G	0.2	rs2462049	0.1159	0.0249	T	g	3.31E−06	− 0.15531	0.01675
11	9:87844548	− 0.0031	0.015	A	G	0.83	rs1931094	0.1163	0.0253	A	g	4.27E−06	− 0.02666	0.016635
**EIF4EBP2**														
1	1:9425722	0.035	0.015	A	G	0.023	rs10864412	− 0.1334	0.0254	A	g	1.58E−07	− 0.26237	0.012644
2	10:64948684	0.011	0.015	T	C	0.5	rs10733789	− 0.1254	0.0272	T	c	4.07E−06	− 0.08772	0.014308
3	15:58881788	− 0.028	0.054	A	G	0.61	rs72743058	− 0.3839	0.0827	A	g	3.47E−06	0.072936	0.019786
4	15:90454175	0.032	0.065	T	C	0.62	rs79943794	− 0.357	0.0766	T	c	3.16E−06	− 0.08964	0.033151
5	16:1547477	− 0.012	0.026	T	C	0.65	rs2745108	0.1927	0.0402	T	c	1.58E−06	− 0.06227	0.018205
6	16:1846089	− 0.047	0.02	T	C	0.017	rs2575348	0.1527	0.0333	T	c	4.47E−06	− 0.30779	0.017155
7	2:167837496	− 0.035	0.04	T	C	0.39	rs79613514	− 0.3407	0.0738	T	c	3.89E−06	0.10273	0.013784
8	21:21934726	− 0.049	0.074	T	C	0.51	rs17003636	− 0.4607	0.0984	T	c	2.88E−06	0.10636	0.0258
9	3:14547897	0.013	0.053	T	C	0.8	rs113664570	0.2666	0.0584	T	c	4.90E−06	0.048762	0.039521
10	5:152342669	0.026	0.017	T	C	0.13	rs72806713	− 0.1366	0.0296	T	c	3.89E−06	− 0.19034	0.015488
11	7:113007324	− 0.021	0.027	T	C	0.43	rs76802510	− 0.2684	0.0496	T	c	6.46E−08	0.078241	0.01012
12	8:106583124	0.021	0.017	A	G	0.2	rs4734879	0.1343	0.028	A	g	1.62E−06	0.156366	0.016023
**RP-S6K**														
1	11:11949472	− 0.0032	0.015	T	C	0.84	rs1355191	− 0.125	0.0263	T	c	1.95E−06	0.0256	0.0144
2	15:83650787	− 0.016	0.068	A	C	0.82	rs77394885	− 0.5291	0.1122	A	c	2.40E−06	0.03024	0.016517
3	19:54294400	0.003	0.025	A	C	0.91	rs3859503	− 0.1892	0.0333	A	c	1.35E−08	− 0.01586	0.01746
4	19:54305398	− 0.073	0.025	T	G	0.0037	rs148800371	0.1637	0.0327	T	g	5.50E−07	− 0.44594	0.023323
5	19:54320716	− 0.047	0.026	A	G	0.075	rs62143197	0.5347	0.029	A	g	8.13E−76	− 0.0879	0.002364
6	19:54333199	− 0.035	0.036	T	C	0.34	rs117021160	0.2834	0.0433	T	c	6.17E−11	− 0.1235	0.016136
7	20:58296885	− 0.081	0.065	T	C	0.22	rs75688971	− 0.4179	0.0836	T	c	5.75E−07	0.193826	0.024193
8	3:22584610	− 0.073	0.057	T	C	0.2	rs58565824	0.4607	0.0937	T	c	8.91E−07	− 0.15845	0.015308
9	3:27587457	0.053	0.036	T	C	0.14	rs9833044	− 0.319	0.0635	T	c	5.13E−07	− 0.16614	0.012736
10	4:145934089	− 0.043	0.059	T	C	0.46	rs35747952	0.3736	0.0809	T	c	3.89E−06	− 0.1151	0.02494
11	5:101423444	0.031	0.046	T	C	0.51	rs1381968	0.3462	0.0746	T	c	3.55E−06	0.089544	0.017655
12	5:152488248	− 0.013	0.014	A	G	0.38	rs62398809	0.1129	0.0247	A	g	4.90E−06	− 0.11515	0.015377
13	5:39170760	0.042	0.076	A	G	0.58	rs148897689	0.4496	0.0943	A	g	1.91E−06	0.093416	0.028574
14	6:68218559	− 0.053	0.074	A	G	0.48	rs72881486	0.3633	0.0745	A	g	1.07E−06	− 0.14588	0.041489
15	7:58384	0.025	0.02	A	C	0.21	rs79777011	0.1634	0.0346	A	c	2.29E−06	0.152999	0.014982
16	8:56752146	0.0069	0.015	A	G	0.65	rs7017005	0.1213	0.0256	A	g	2.14E−06	0.056884	0.015292

Association between genetically determined EIF-4E, EIF-4A, EIF-4G, EIF4EBP2, and RP-S6K, mTOR downstream targets with Diabetes health outcome. The data source for exposure is the human plasma proteomics–GWAS INTERVAL study (n = 3,301) participants from publically available aggregate summary data^47^. The source for diabetes health outcome is DIAGRAM database (DIAbetes Genetics Replication And Metaanalysis case (n = 26,676), and control (n = 132,532); https://www.diagram-consortium.org/. For sensitivity analysis, we used uncorrelated SNPs for EIF-4E, IF-4A, EIF-4G, EIF4EBP2, and RP-S6K. Mendelian Randomization (MR-Base) R package was used in the analysis.

## Discussion

**Figure 5:**
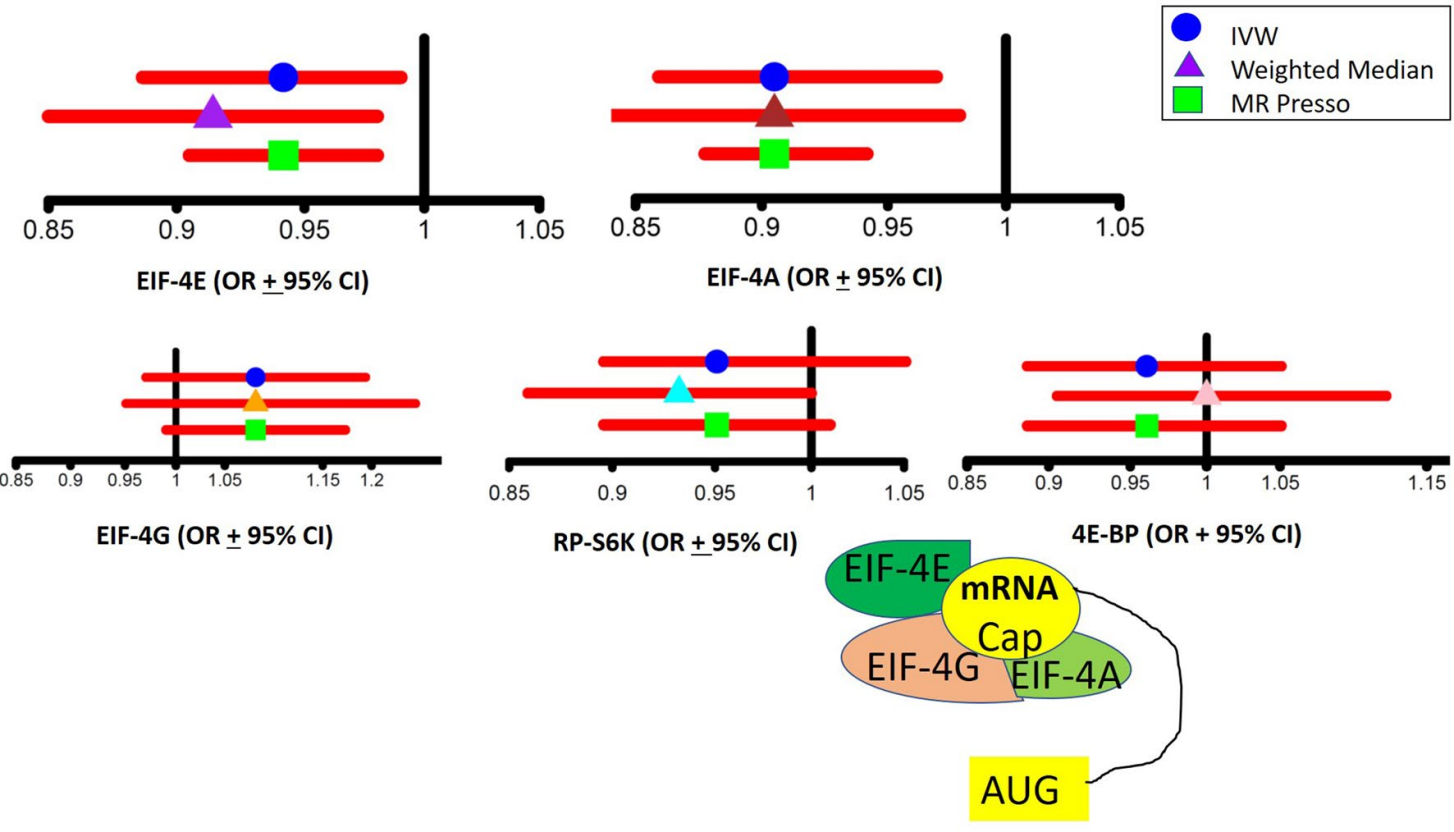
Comparisons of the Odds Ratio values and the 95% Upper Confidence Level and Lower Confidence Level between different MR Models. Comparison of the overall estimates of odds ratio values obtained by different MR models, including Inverse Variance Weighted (IVW), Weighted Median (WM), and MR PRESSO analyses of genetically determined EIF-4E, EIF-4A, EIF4G, EIF4EBP, and RP-S6K. Mendelian Randomization (MR-Base) R package and MR Base web applications were used in the analysis^58^.

To our knowledge, this is the first study to leverage MR to determine the causal association of EIF-4E and EIF-4A circulating levels with diabetes mellitus in humans. The data suggest that higher circulating levels of EIF-4E (OR 0.94, CI [0.88, 0.99]) and EIF-4A (OR 0.90, CI [0.85, 0.97]) translation initiation proteins may be causally associated with a lower risk of type 2 diabetes mellitus. As shown in the leave-one-out sensitivity IVW analysis, the effect size for EIF-4E is − 0.06 (OR 0.94), and for EIF-4A is − 0.10 (OR 0.90) (Supplemental Figure 2). Per our study objectives, five mTORC1 downstream targets (RP-S6K, EIF4EBP2, EIF-4G3, EIF-4E cap-dependent translation, and EIF-4A cap-dependent translation factor), circulating levels were instrumented by strongly associated SNPs (Fig. 5). This unbiased MR approach found that EIF-4E and EIF-4A, which are essential for 5′-cap-dependent mRNA translation, could have a causal protective association with type 2 diabetes. The directionality of the MR association between circulating levels of EIF-4E and EIF-4A, mTORC1-dependent proteins, and type-2 diabetes mellitus was protection, i.e., higher circulating levels of EIF-4E and EIF-4A may be causally associated with decreased risk of type 2 diabetes mellitus. This data is consistent with experimental data in mTORC1 knockout mice showing an essential role of mTORC1 in beta cell functions and insulin processing^45^. Our data indicated that the risk of diabetes varied with mTOR downstream target EIF-4E and EIF-4A, but not with RP-S6K or EIF4EBP2. The association for both EIF-4E cap-dependent translation factor and EIF-4A were robust to Bonferroni correction.

In this study, we used the two-sample MR, which increases the sample size and provides unconfounded estimates. However, MR requires several stringent assumptions to be fulfilled. First, the genetic predictors should be strongly associated with the exposures (IV1); to satisfy this assumption, we selected only the SNPs strongly associated with EIF-4E, EIF-4A, EIF-4G, EIF4EBP2, and RP-S6K circulating plasma levels. Second, no confounders of the genetic instrument on outcome should exist, which we assume from genetic randomization at conception (IV2). The underlying genetic studies were controlled for population stratification when necessary. Third, genetic predictors of exposure should not be independently associated with diabetes (IV3), and only mediate their effects via the relevant exposure. In other words, there is little evidence of pleiotropy. Sensitivity analyses with different assumptions about pleiotropy gave estimates with the IVW (Fig. 5). Since this study only used aggregate summary statistics, we were not able to assess differences by age, gender, or other factors. However, causal effects are generally expected to act consistently.

In contrast to RCTs, MR studies inform the role of a specific causal pathway but do not reveal whether an intervention will be efficient. Furthermore, RCTs only report the effects of short-time exposures, while MR studies usually identify the lifetime exposures. The limitations of the MR study are those cohort populations recruited for the exposure (INTERVAL Study), and outcomes (DIAGRAM Study) were from European descent, which may limit the generalizability of the results to other populations.

Cell culture and animal studies have documented an integral role of mTORC1 in glucose metabolism and diabetes^24,25,67–70^. Blandino-Rosano and colleagues have shown in Raptor, a mTORC1 specific partner, in a conditional knockout mouse model led to pancreatic β-cell failure due to defects in insulin secretion and cell proliferation, and thus furthered the development of diabetes^44–46,71,72^. Ardestani and colleagues reported that mTORC1 positively regulates beta-cell growth and proliferation, thus promoting insulin secretion, while the hyperactivation of mTORC1 led to beta-cell failure in type 2 diabetes^16^. Similarly, Tuo and Xiang reported a paradoxical role of mTORC1 on insulin secretion and β-cell metabolism^31^. The biphasic response of pancreatic β cells to mTORC1 activation has also been reported in mice^43^. Shigeyama and colleagues found that ablated Tuberous Sclerosis Complex 2 (TSC2), an upstream inhibitor of mTORC1m in mice (βTSC^−/−^ mice), led to an initial increase in β cell mass and size in an mTOR-dependent manner. Furthermore, the mice were hyperinsulinemic and hypoglycemic. However, after 40 weeks of age, these mice became hypoinsulinemic and hyperglycemic due to a reduction in β cell mass and number^43^. Taken together, these two observations suggest that mTOR-mediated a biphasic response on insulin secretion.

Similarly, a biphasic insulin sensitivity to mTOR inhibitor, rapamycin, has been reported in C2C12 myocytes^37^. Reciprocal regulation and cross-talk between mTORC1 and mTORC2 have been suggested^35,73^. Further, a distinctive role of mTORC2 in regulating insulin secretion and pancreatic β cell functions has also been reported^18,35^. Blandino-Rosano and colleagues found that the deletion of EIF-4EBP2 induced feedback and elevated glucose tolerance by increasing pancreatic beta-cell mass in knockout mice^74^. It is possible that EIF-4E and EIF-4A may regulate the mRNA translation of insulin protein, insulin receptor, or the enzymes involved in the regulation of glucose metabolism such as those regulating glycolysis and TCA cycle. We leveraged the use of the PhenoScanner curated database to explore possible causal mechanistic associations between the circulating levels of EIF-4E and EIF-4A and type 2 diabetes mellitus. We found 3 EIF-4E proxy SNPs namely, rs11084300, rs3848582, rs62143198, and 4 EIF-4A predicting SNPs, namely, rs3859507, rs11084300, rs34436714, rs146117463, that are associated with the NLRP12 gene. The NLPR12 is an inhibitory innate immune sensor and a marker of monocytes and macrophage inflammasomes. Recently, NLPR12 has been implicated in protecting against obesity by regulating the gut microbiota homeostasis, insulin-tolerance, and inflammation in mice^75^. Thus, we propose that EIF-4E and EIF-4A translation initiation proteins may have a protective effect on type 2 diabetes that is possibly mediated via NLPR12 inflammasome, which regulates the gut microbiome. Experimental laboratory data is warranted to explore the mechanistic pathway for this effect.

## Conclusion

This unbiased MR study is consistent with a causal protective association of EIF-4E and EIF-4A with type 2 diabetes. EIF-4E and EIF-4A circulating levels, critical regulators of gene translation and protein functions, may be targets for intervention by repurposing existing therapeutics to reduce the risk of type 2 diabetes.

## Supplementary information

Supplementary Information 1.

## Data Availability

Data on diabetes health outcomes were obtained publically available genetic associations with diabetes from the DIAbestes Genetics Replication And Meta-analysis (DIAGRAM) case–control study https://www.diagram-consortium.org/download. Data about mTOR-related gene exposure were obtained from the publically available INTERVAL study Proteomics-GWAS data in 3,301 participants from the INTERVAL study, which included ~ 3,600 plasma protein assay https://www.phpc.cam.ac.uk/ceu/proteins/ (Sun et al., Nature, 2018^39^). The authors would like to thank all investigators for sharing the data.

## Abbreviations

SNPs: Single nucleotide polymorphism
mTOR: Mammalian target of rapamycin
EIF-4E: Eukaryotic translation initiation factor 4E
EIF4EBP2: Eukaryotic translation initiation factor 4E binding protein 2 (4E-BP)
mTORC1: Mammalian target of rapamycin complex 1
mTORC2: Mammalian target of rapamycin complex 2
GWAS: Genome-wide association studies
IVW: Inverse variance weighted
WM: Weighted median
MR: Mendelian randomization
RP-S6K: Ribosomal protein S6K kinase
