# Supplementary material for: Causal association between mTOR-dependent EIF-4E and EIF-4A circulating protein levels and type 2 diabetes: a Mendelian randomization study
Source: Sci Rep. 2020 Sep 25;10:15737. doi: 10.1038/s41598-020-71987-8 (PMC7519073; doi:10.1038/s41598-020-71987-8)
Supplement: Supplementary file 1 — Supplementary Information 1. [file 41598_2020_71987_MOESM1_ESM.pdf]

Supplemental Material

Causal association between mTOR-Dependent EIF-4E and EIF-4A circulating protein levels and Type 2 Diabetes: A Mendelian Randomization Study

Ghada A. Soliman[1, 2*] and C. Mary Schooling[1, 3],

[1] Department of Environmental, Occupational and Geospatial Health Sciences, The City University of New York, Graduate School of Public Health and Health Policy, 55 West 125th St, New York, NY 10027, USA

[3]School of Public Health, Li Ka Shing Faculty of Medicine, The University of Hong Kong, 7 Sassoon Road, Hong Kong, China.

[2*]Address correspondence to Ghada A. Soliman (email: ghada.soliman@sph.cuny.edu)

SUPPLEMENTAL TABLES

Supplemental Table S1: SNPs associated with exposure to mTOR targets:

**Protein**	SNP	Beta	SE	Effect	Other	p-Value	Chrpos
**EIF-4E**	rsID	Exposure	Exposure	Allele	Allele	Exposure	Exposure
1	rs2209485	0.1637	0.034	t	c	1.51E-06	10:20905192
2	rs74842834	0.4443	0.0947	t	g	2.69E-06	11:23204504
3	rs192206210	-0.5846	0.1211	t	g	1.41E-06	15:87118467
4	rs138236097	-0.2673	0.0583	a	g	4.47E-06	16:47761066
5	rs741454	-0.1426	0.031	t	c	4.17E-06	19:29350614
6	rs11084300	0.1585	0.0272	t	c	5.75E-09	19:54311090
7	rs3848582	-0.1776	0.0364	a	g	1.07E-06	19:54317794
8	rs62143198	0.4669	0.0296	a	g	6.76E-56	19:54320939
9	rs149036167	0.5168	0.108	t	c	1.70E-06	2:119720418
10	rs62130614	0.4606	0.0999	t	g	3.98E-06	2:29505824
11	rs113679228	0.3464	0.0732	a	g	2.24E-06	4:138007589
12	rs12640699	0.1574	0.0341	a	c	4.07E-06	4:167757172
13	rs142569846	0.2303	0.0468	t	g	8.71E-07	5:116455931
14	rs192028145	0.5295	0.1025	a	g	2.40E-07	5:66685975
15	rs116934738	-0.3758	0.077	t	c	1.05E-06	8:87691869
16	rs2772577	0.1361	0.0292	a	g	3.02E-06	9:24547232
**EIF-4A**	SNP	Beta	SE	Effect	Other	p-Value	Chrpos
	rsID	Exposure	Exposure	Allele	Allele	Exposure	Exposure
1	rs74512707	0.1785	0.0385	a	g	3.55E-06	12:95350637
2	rs151270869	0.4543	0.0938	a	g	1.29E-06	15:86409988
3	rs3859507	0.1777	0.0327	t	g	5.75E-08	19:54295370
4	rs11084300	0.1431	0.0272	t	c	1.48E-07	19:54311090
5	rs34436714	0.4687	0.0291	a	c	2.00E-58	19:54327313
6	rs146117463	-0.2062	0.042	a	g	9.33E-07	19:54329672
7	rs34131899	-0.2829	0.0591	a	g	1.70E-06	2:29058128
8	rs6792693	-0.134	0.0286	a	g	2.82E-06	3:27245225
9	rs1447676	-0.1182	0.0257	t	c	4.37E-06	3:74143047
10	rs2462049	0.1159	0.0249	t	g	3.31E-06	7:85099898
11	rs1931094	0.1163	0.0253	a	g	4.27E-06	9:87844548
**EIF-4G3**	SNP	Beta	SE	Effect	Other	p-Value	Chrpos
	rsID	Exposure	Exposure	Allele	Allele	Exposure	Exposure
1	rs7955609	0.1251	0.0263	a	g	2.00E-06	12:116387486
2	rs112309230	0.5745	0.1158	t	c	6.92E-07	12:127417047
3	rs143862167	-0.204	0.044	t	c	3.55E-06	12:39641451
4	rs704	0.1618	0.0245	a	g	4.27E-11	17:26694861
5	rs142978915	0.4055	0.0885	t	c	4.57E-06	3:53892603
6	rs1411879	0.4092	0.089	a	g	4.27E-06	9:3762150

Supplemental Table 1 SNPs associated with exposure to mTOR targets—Continued

**EIF-4EBP2**	SNP	Beta	SE	Effect	Other	p-Value	Chrpos
	rsID	Exposure	Exposure	Allele	Allele	Exposure	Exposure
1	rs10864412	-0.1334	0.0254	a	g	1.58E-07	1:9425722
2	rs10733789	-0.1254	0.0272	t	c	4.07E-06	10:64948684
3	rs72743058	-0.3839	0.0827	a	g	3.47E-06	15:58881788
4	rs79943794	-0.357	0.0766	t	c	3.16E-06	15:90454175
5	rs2745108	0.1927	0.0402	t	c	1.58E-06	16:1547477
6	rs2575348	0.1527	0.0333	t	c	4.47E-06	16:1846089
7	rs79613514	-0.3407	0.0738	t	c	3.89E-06	2:167837496
8	rs17003636	-0.4607	0.0984	t	c	2.88E-06	21:21934726
9	rs113664570	0.2666	0.0584	t	c	4.90E-06	3:14547897
10	rs72806713	-0.1366	0.0296	t	c	3.89E-06	5:152342669
11	rs76802510	-0.2684	0.0496	t	c	6.46E-08	7:113007324
12	rs4734879	0.1343	0.028	a	g	1.62E-06	8:106583124
**RP-S6K**	SNP	Beta	SE	Effect	Other	p-Value	Chrpos
	rsID	Exposure	Exposure	Allele	Allele	Exposure	Exposure
1	rs1355191	-0.125	0.0263	t	c	1.95E-06	11:119496472
2	rs77394885	-0.5291	0.1122	a	c	2.40E-06	15:83650787
3	rs3859503	-0.1892	0.0333	a	c	1.35E-08	19:54294400
4	rs148800371	0.1637	0.0327	t	g	5.50E-07	19:54305398
5	rs62143197	0.5347	0.029	a	g	8.13E-76	19:54320716
6	rs117021160	0.2834	0.0433	t	c	6.17E-11	19:54333199
7	rs75688971	-0.4179	0.0836	t	c	5.75E-07	20:58296885
8	rs58565824	0.4607	0.0937	t	c	8.91E-07	3:22584610
9	rs9833044	-0.319	0.0635	t	c	5.13E-07	3:27587457
10	rs35747952	0.3736	0.0809	t	c	3.89E-06	4:145934089
11	rs1381968	0.3462	0.0746	t	c	3.55E-06	5:101423444
12	rs62398809	0.1129	0.0247	a	g	4.90E-06	5:152488248
13	rs148897689	0.4496	0.0943	a	g	1.91E-06	5:39170760
14	rs72881486	0.3633	0.0745	a	g	1.07E-06	6:68218559
15	rs79777011	0.1634	0.0346	a	c	2.29E-06	7:58384
16	rs7017005	0.1213	0.0256	a	g	2.14E-06	8:56752146

SUPPLEMENTAL TABLE S2

Table S2 Supplement

T-Distribution and F-Statistics of the Beta. Exposure Variable on Diabetes Health Outcome

Exposure ID	Causal Estimate (beta)	SD	T-Stat	P-value	MR-PRESSO Global
EIF-4E	-0.06552	0.02219	-2.95269	0.00987	0.9053
EIF-4A	-0.09210	0.02039	-4.51705	0.00195	0.9885
EIF-4G3	0.07317	0.04368	1.67513	0.15475	0.6732
EIF-4EBP	-0.04147	0.04646	-0.89259	-0.39118	0.1056
RP-S6K	-0.05583	0.03157	-1.76819	0.09735	0.2552

SUPPLEMENTAL FIGURES

Supplemental Figure S1: Scatter Plot Analysis and Comparison Between MR Models

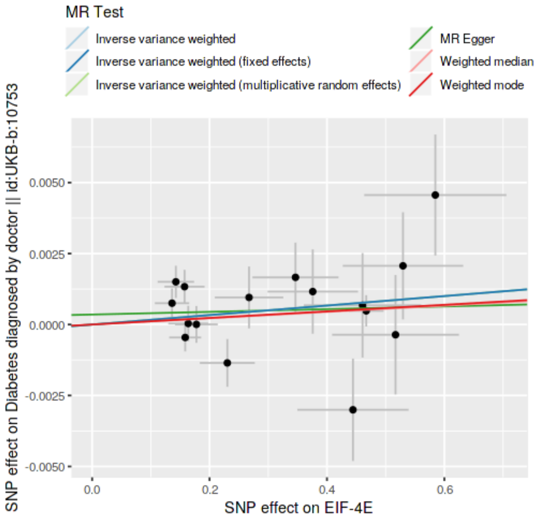

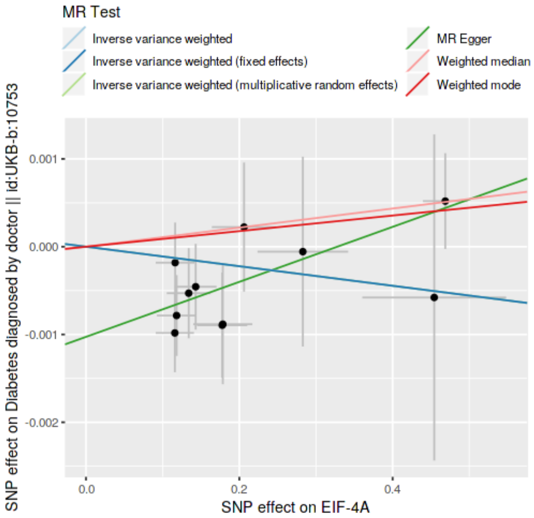

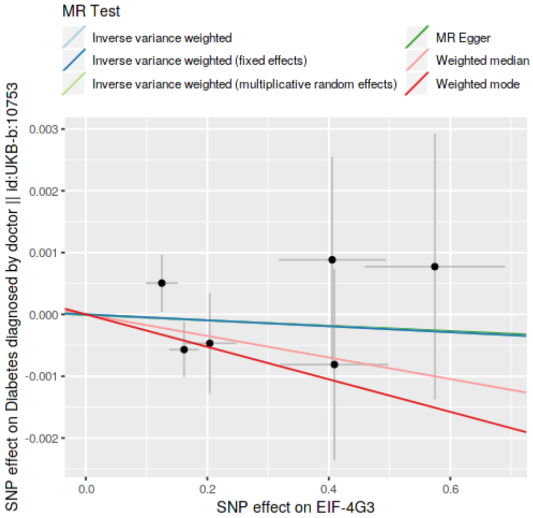

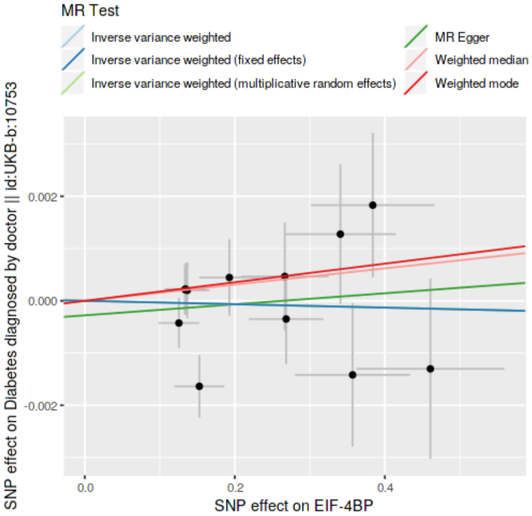

29

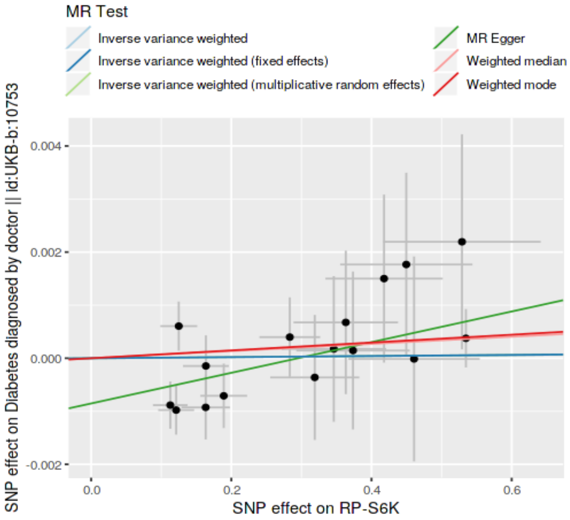

Supplemental Figure 1: SNP effects on the outcome (y-axis) plotted against SNP effects on the exposure (x-axis). The slopes of the lines represent the causal association, and each model has a different line. MR-Base R web-based application was used in the analysis. MR Egger estimate does not automatically pass through the zero origins.

Supplemental Figure 2: Leave-One-Out- Sensitivity Analysis

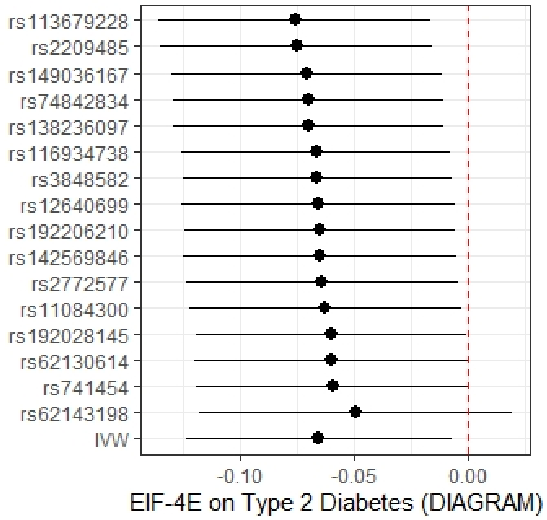

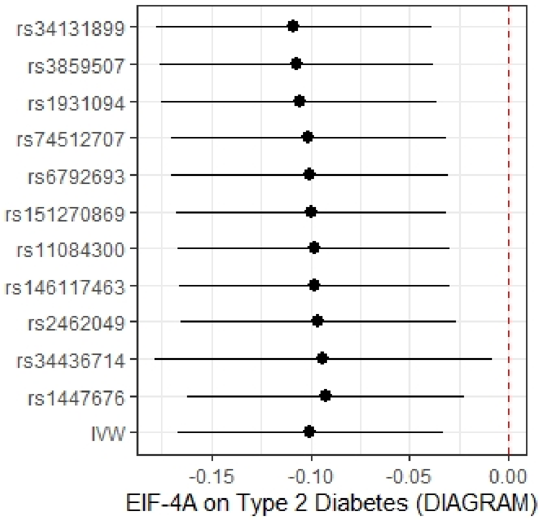

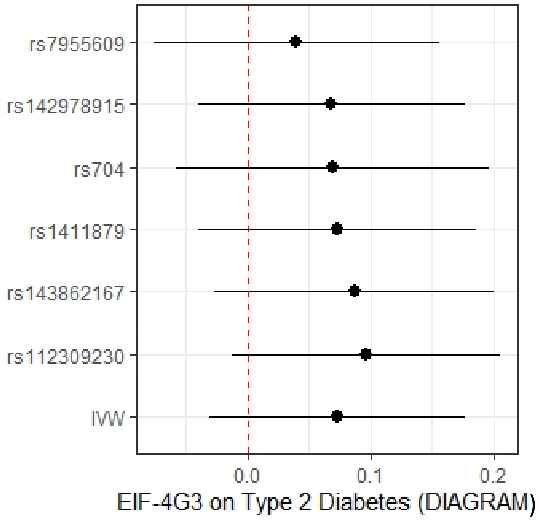

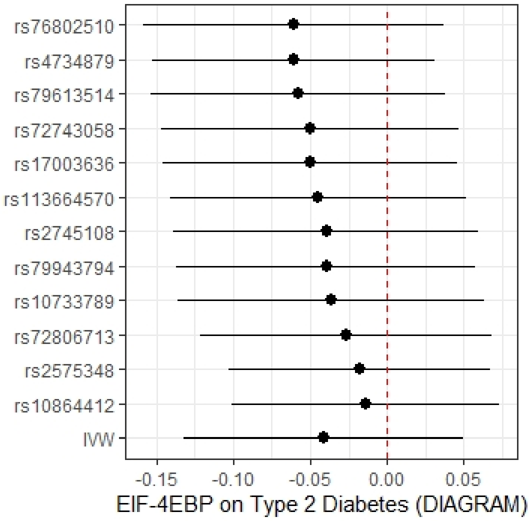

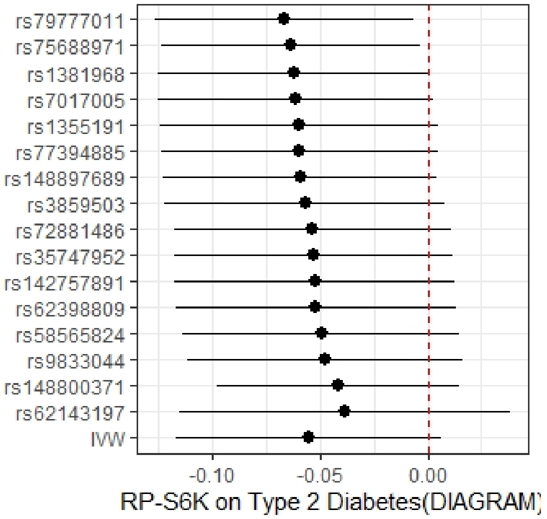

Supplemental Figure 2: Results of the leave-one-out method in MR sensitivity analysis. The vertical dotted red line denotes the null effect in each panel. The y-axis represents the OR and CI of the SNPs without the one-SNP indicated on the axis. The x-axis represents the effect size of the corresponding exposure. The Inverse Variance Weighted (IVW) denotes the overall effect size estimate.

SUPPLEMENTAL FIGURE 3: Funnel plot Comparison Between MR Methods

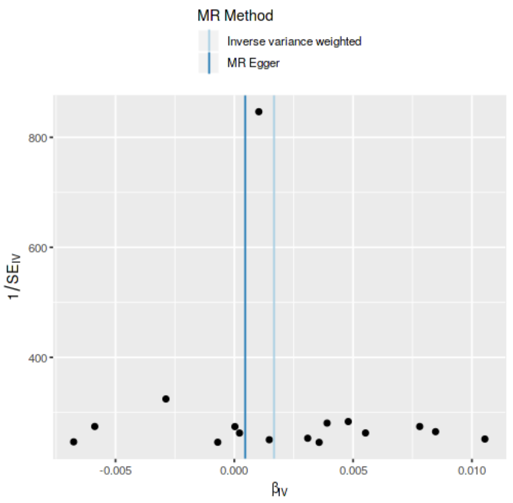

Funnel plot analysis for EIF-4E

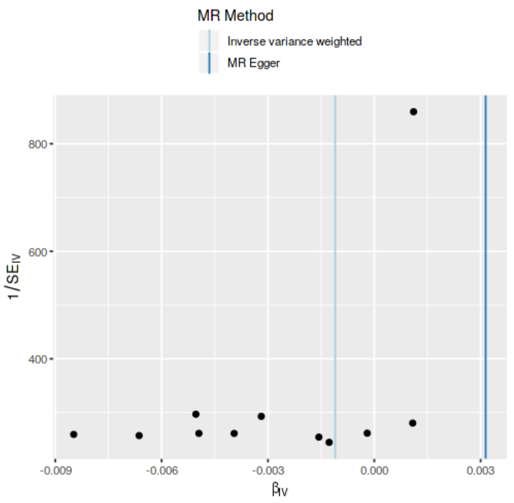

Funnel plot analysis for EIF-4A

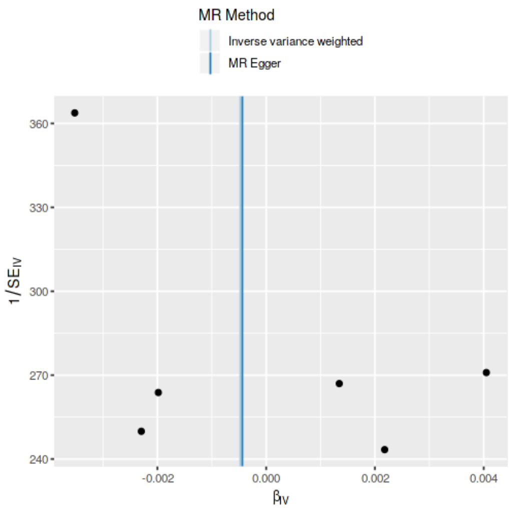

Funnel plot analysis for EIF-4G3

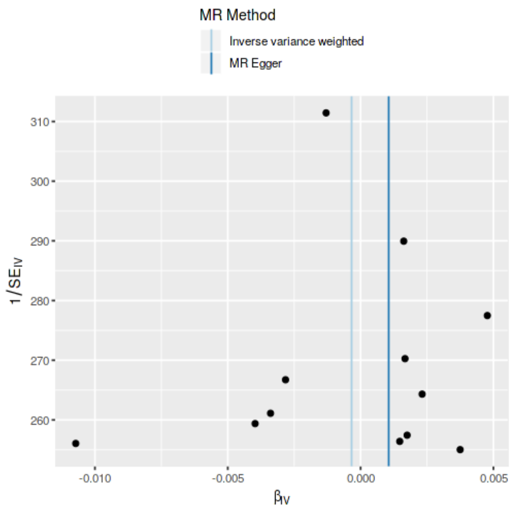

Funnel plot analysis for EIF-4E-BP

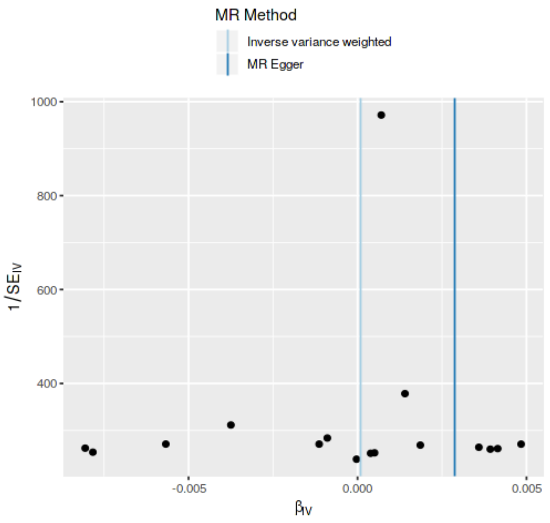

Funnel plot analysis for RP-S6K

Supplemental Figure 3: Funnel plot analysis to assess heterogeneity. The vertical lines represent the causal estimates using all SNPs combined into a single instrument for each of the MR models (IVW, and MR Egger). The x-axis represents the causal estimate, and the y-axis represents the inverse of the instrument strength. MR-Base R package and web application were used in the analysis.
